# Average Carbohydrate and CaLoric Intake of Patients with Type-2 diabetes at a tertiary care hospital in Pakistan: ACCLIP study

**DOI:** 10.12669/pjms.40.9.8968

**Published:** 2024-10

**Authors:** Kiran Habib, Shumaila Gul, Azizul Hasan Aamir

**Affiliations:** 1 Kiran Habib, M.Sc Department of Diabetes, Endocrinology and Metabolic Diseases, MTI Hayatabad Medical Complex, Peshawar, Pakistan; 2 Shumaila Gul, M.Sc (Hons) Department of Diabetes, Endocrinology and Metabolic Diseases, MTI Hayatabad Medical Complex, Peshawar, Pakistan; 3 Azizul Hasan Aamir, MRCP, FRCP, FACE HOD/Professor, Khyber Girls Medical College, Hayatabad Medical Complex, Peshawar, Pakistan

**Keywords:** Type-2 diabetes mellitus, BMI, Lifestyle, Carbohydrate intake, Caloric intake

## Abstract

**Background & Objective:**

Type-2 Diabetes Mellitus (T2DM) is one of the most common chronic non-communicable diseases and a serious health issue worldwide because of its rising prevalence amongst the young adults. Dietary diversity, rapid economic development and sedentary lifestyle are amongst the common factors contributing for the rapid rise of diabetes. Our objective was to assess the average carbohydrate (CHO) and caloric consumption and its association with obesity and disease status in patients with Type-2 diabetic patients in an outpatient setting.

**Methods:**

This study was performed at an outpatient department (OPD), of Hayatabad Medical Complex, Peshawar. Patients with T2DM were interviewed who completed dietary assessment using 24 hours dietary recall method.

**Results:**

A total of 150 patients with Type-2 diabetes were interviewed. The mean carbohydrate intake was 400.3±106 mg/day, out of which 43.3 % participant’s had carbohydrate intake above recommendations. The mean energy intake for all participants was 2504.5±587.4 Kcal/day. Majority of the participants were overweight and obese with mean BMI of 28kg/m^2^ ± 4.4. There was no significant difference in energy and carbohydrate intake between male and female participants.

**Conclusions:**

Majority of Pakistani patients with Type-2 diabetes consume foods rich in carbohydrate as well as have high caloric value. These finding were more in patients with no formal education compared to those who were well educated with a degree.

## INTRODUCTION

Type-2 Diabetes Mellitus (T2DM) is a metabolic disease with the primary pathophysiological abnormalities including insulin resistance, relative insulin deficiency and decreased incretin action. The most common risk factors include sedentary lifestyle, obesity, positive family history and positive history of gestational diabetes.^1^ The prevalence of Type-2 diabetes mellitus is 16.98% in Pakistan, and the prevalence of prediabetes is 10.91%. This alarming trend in diabetes prevalence demands comprehensive measures for an early diagnosis, treatment, and prevention of T2DM at the community level.^2^ Poor diabetic control is directly linked to both macro and microvascular complications and similarly well controlled diabetes results in reduction of complications.^3^

In Pakistan, patients with T2DM generally lack enough information on the important role of lifestyle and dietary changes in glycemic control primarily due to shortage of trained dieticians, and diabetes educators in the primary or secondary health care systems. There is an utmost need to educate people in Pakistan regarding the disease and the different management options available particularly, lifestyle changes and dietary changes required for a patient with Type-2 diabetes. Carbohydrate and caloric intakes of Pakistani patients with type T2DM has not been studied in the past. Hence, our study was aimed to evaluate the average CHO and caloric intake of Type-2 diabetic patients and its association with obesity and control of diabetes. This information is very much required to identify the trends of CHO and caloric consumption of patients with diabetes for a better understanding of what dietary modifications are required in managing diabetes mellitus in Pakistani setup.

## METHODS

This study was performed at an outpatient department (OPD), of Hayatabad Medical Complex, Peshawar, which is a tertiary care center with trained dietician. All the participants who were diagnosed with Type-2 diabetes from August 2023 to October 2023were included in the study regardless of the duration of diabetes. Informed consent was taken from all the participants.

A standard and preprinted questionnaire was used by two trained interviewers which included questions regarding demographic data, onset of diabetes, use of oral antidiabetics and injectables. Patients were asked if they had any formal session regarding dietary education with dietitian in the past. Sample size was calculated on the WHO sample size calculator, a total of 150 subjects were chosen based on a prevalence of 16.98% Type-2 diabetes with 95% confidence interval and 5% margin of error.^2^

### Ethical Approval:

The study got approval from ethical review committee (Ethical approval no.1503), Date June 09, 2022.

Obesity was categorized as up to BMI of 23 (Group-1), 23-25 (Group-2) more than 25-29.9 (Group-3) and (Group-4) with BMI of 30 and above. HbA1c and Random Blood Glucose (RBG) levels of the participants were recorded at the time of interview. HbA1c was categorized as less than 7% (Group-1), from 7-10% (Group-2) and HbA1c more than 10% as (Group-3). Similarly, RBG levels was categorized as less than 140mg/dl (Group-1), 140-180mg/dl (Group-2), 180-250 mg/dl (Group-3) and more than 250mg/dl (Group-4).

For assessing dietary intake, 24-hours dietary recall method was used. Participants were asked to recall what they ate during the last 24-hours from the previous morning till the morning of interview. They were asked to mention portion sizes of food by showing them photos of different measured plates, cups, and spoons. The data gathered from 24-hours dietary recall was converted to grams and then the total intake of calories and CHO intake was calculated. The nutritional composition of ingredients was computed by using the food composition table for Pakistan (2001). 4

### Statistical analysis:

Statistical analyses were carried out by using SPSS packages version 16. All data was presented as means ± standard deviation and counts (percentages) for categorical variables, while differences in the major characteristics between participants were examined by independent sample *t*-tests and One Way ANOVA. All *P*-values were two-sided, and the significance level was 0.05.

## RESULTS

A total of 150 patients with T2DM were included in the study. Out of the 150 participants 75(50%) were male. The mean age of the participants was 50.3±9.5 years. The socio-economic distribution of the respondents showed that majority 78.7% were not formally educated, with most female (49.3%) as house wife, (Table-I).

**Table-I:** Socio-demographic characteristics of study participant.

Characteristics		Frequency(N)	%	Mean ± SD
Gender	Male	75	50	_
	Female	75	50	
Age	<40 Years	19	12.7	50.3 ± 9.5
	40 – 60 years	113	75.3	
	>60 years	18	12	
Education	No formal education	118	78.7	_
	Secondary school	22	14.7	
	Postgraduate	10	6.7	
Occupation	Housewife	74	49.3	_
	Unemployed	50	33.3	
	Common Job	26	17.3	
BMI	≤ 23	19	12.7	28 ± 4.4
	>23 up to 25	26	17.3	
	>25 up to 29.9	56	37.3	
	30 and above	49	32.7	

The mean BMI was 28kg/m^2,^ among 150 patients’ majority were overweight and obese, and only 12.7% had a normal nutritional status as measured by their (BMI) Body mass index, (Table-I) Out of 150 participants, majority (47.3%) had HbA1c greater than 10% while (37.3%) had between 7-10% and only (2%) had less than 7%, while HbA1c data was missing in 20 patients. Majority of the participants (48.7%) were having RBG more than 250mg/dl, while only (5.3%) have RBG less than 140mg/dl. The mean RBG level was 292mg/dl (SD ±97.7) Most of the patients 42.7% were on insulin + Oral Anti Diabetics (OADs), also majority (53.3%) we’re not previously counselled by formal dietitian about dietary restrictions and lifestyle modifications.

### Carbohydrate and caloric intake of patients:

The mean daily CHO intake was 400.3gm without a significant difference between males and females (P>0.05). Carbohydrate intake was significantly high 418.7gm (SD 97.9) among those who were overweight ant obese (P = 0.04) over those with the BMI of normal range. We did not observe any significance in energy and CHO intake of the participants according to educational status (P >0.05). However, there was a significant difference between those who were previously counselled by dietitian consuming comparatively less calories (2321.2kcal) and CHO (367gm) than those who were not.

The mean total daily energy intake of the participants was 2504.5kcal (SD 587.4) with men consuming more energy than women, this difference is however not statistically significant (P >0.05). The caloric intake was significantly high 2609kcal (SD 557) among those who were overweight and obese (P = 0.01) over those with BMI below25kg/m^2^ (Table-II). Majority of the participants (56%) were consuming carbohydrates within the recommendations i.e., 45-65% while 43.3% were consuming CHO above recommendation i.e., >65% and only (.7%) recorded below recommended intake of CHO/day, (Table-III). There was a significant relationship between HbA1c and RBG levels with caloric intake of the participants (p <0.05). The RBG was significantly higher in patients with high CHO intake. Numerically, the HbA1c level increases with an increase in CHO intake, however statistically the values were not significant (p = 0.07).

**Table-II:** Average Energy and carbohydrate intake of participants.

Characteristics		Energy intake			Carbohydrate intake		

		Mean (kcal)	SD	p-value	Mean (gm)	SD	p-value
GENDER	Male	2579.0	516.8	.121	404.5	88.1	.265
	Female	2430.0	645.3		389.6	112.0	
BMI	≤23	2299.2	596.1	.001	369.7	105.1	.004
	23 - 25	2127.5	408.7		348.5	119.4	
	>25	2609.0	557.0		418.7	97.9	
Educational status	No formal education	2499.5	596.4	.840	405.0	105.8	.597
	Sec. School	2483.7	411.4		383.5	71.8	
	Postgraduate	2336.7	578.7		333.6	68.0	
Previously counselled by dietitian	Yes	2321.2	446.6	.002	367.0	80.2	.003
	No	2630.8	627.4		423.4	109.6	
Overall Mean±SD		2504.5	587.4		400.3	106.0	-

**Table-III:** Percentage of carbohydrate (CHO) and its relationship to recommended intakes.

Recommendations		N	%
Below recommendations i.e	<45% CHO INTAKE	1	.7
Within recommendations i.e	45 – 65% CHO INTAKE	84	56.0
Above recommendations i.e	>65% CHO INTAKE	65	43.3

**Table-IV:** Energy and carbohydrate intake relation with glycemic control.

Characteristics	HbA1c		RBS	

	Mean	p-value	Mean	p-value
***Caloric intake***		.001		.000
<1800	8.5		247	
1800 – 2000	10.5		228	
2000 – 2500	10.7		280	
>2500	11.0		327	
***Carbs intake***		.070		.000
<250	8.8		278	
250 – 350	10.1		235	
350 – 450	10.8		303	
>450	11		335	

## DISCUSSION

In our study majority of participants were either overweight or obese. Obesity is one of the leading risk factors for the development of T2DM. Management of obesity and overweight is important for better glycemic control and prevention of complications. Although with the advancement in management of diabetes and availability of newer, potent OADs and injectables, but dietary management still remains to be a corner stone in the management of T2DM.^5^ In dietary therapy, diet composition, amount, distribution, and time of food intake are important factors. Higher CHO and energy consumption is frequent among patients with diabetes, and this is due to use of wheat bread and rice which is the staple food of our population.^6^ For obese Type-2 diabetic patients, minimum 5% weight loss is desirable which can only be possible by reducing energy dense diets.^7^ Energy-dense diets may contribute to insulin resistance by their higher levels of saturated fats, and refined carbohydrate. It must be decreased for a viable therapeutic option for patients with Type-2 diabetes and obesity.^8^

A study by Siddiqui M et al.^9^ suggests that Pakistani diet is typically energy dense with a higher percentage of saturated fats, and free sugar contributing to high calories intake. Use of ghee, high intake of meat, judicious use of sweets in feasting events of life is common dietary practices in Pakistan. In our study we observed that there is an increase intake of CHO and calories of patients with T2DM, where men consumed more carbohydrate and calories than women, however it was not statistically significant. Unfortunately, there are no other studies, which quantified the CHO, and caloric intakes of Pakistani diabetic patients for comparison.

A survey study by Shahabuddin S et al.^10^ on relation of diabetes with people’s lifestyle in small cities of Bangladesh suggests that majority of the patients 84.8% are lacking healthy diet and consumption of carbohydrate intake is higher which leads to a raise of high blood sugar levels. There was more responsible eating habits and adherence to dietary restrictions with the advancing socio-economic status. Also, on the same lines a study by Robat Sarpooshi D et al.^11^ in Iran concluded that High education levels facilitate adherence to self-care behavior whereas low education levels make this process difficult.

Korean study by Kim SH et al.^12^ established the daily CHO and caloric intake of patients with T2DM to be 1,757.5 kcal/day, and 60.9% of total intake respectively. Men had a significantly higher intake of energy and a lower intake of CHO while in the obese women, CHO intake was higher. Grylls W et al.^13^ identified that reducing dietary saturated fat and excess body weight may help with glycemic control, whereas decreasing dietary CHO energy intake may help with weight management. A study by Psaltopoulou T et al.^14^ showed that obesity can significantly raise the chance of developing diabetes, although losing weight can also significantly lower plasma glucose levels. Our study showed over 70% diabetic patients are either overweight or obese, with high carbohydrate and caloric intake causing high blood sugar levels. Therefore, it’s crucial to evaluate energy intake and educate patients on healthy lifestyle modifications.

Rivellese AA et al.^15^ assess the average calorie intake of patients with T2DM. The mean calorie intake was 1725±497 kcal (1800 for men, 1610 for women), in Italy. Compared to these values, Pakistani diabetic patients seem to consume high CHO and energy dense diet. American association of clinical Endocrinologists recommend carbohydrates to contribute 45-65% of the total energy intake.^16^ In our study 56% fulfilled the recommendation for CHO intake while 43.3% exceeded it. Proportion of energy intake from carbohydrate is generally low among western patients and high among those of Asian origin where main staple food is wheat and rice. In a study by Mahmood K and Aamir AH^17^ found that majority of the Pakistani patients with Type-2 diabetes had poor glycemic control with mean HbA1c of 10.183 ± 1.73 SD. In our study glycemic control of patients was also poor with the mean HbA1c of 10.6% ±1.96 and mean RBG level of 292 mg/dl±97.7.

The American Dietetic Association reviewed 18 studies that involved the provision of MNT as part of treatment for diabetes which is an effective and increasingly reasonable approach to control as well as prevent T2DM.^18^ In our study the CHO and caloric intake of patients who did not receive any dietary counselling session were high comparatively to those who were previously educated by dietitian. Unfortunately, there is a lack of trained dietitians in our public and private setups, hence there is a lack of awareness about dietary and lifestyle modifications amongst our patients. All these factors contribute towards high CHO and caloric intake and ultimately towards uncontrolled diabetes and obesity. The knowledge about dietary requirements of every patient should be given individually with clear purpose, so that they understand and follow it in practice particularly regarding CHO and caloric intake. Patient education programs and awareness campaign should be integrated in health care system.

### Limitations:

This study has limitations due to missing HbA1c data of 20 participants and it is single centre study with small sample size. As the prevalence of diabetes in Pakistan is high, therefore a large-scale, multi centre study would better understand the findings. This is the first study to calculate the actual CHO and caloric intake of patients with T2DM in Pakistan, and its impact on their glycemic control where no previous data is present.

## CONCLUSION

We conclude that patients with T2DM consume both high caloric as well as food rich in carbohydrates. These finding were more in patients with no formal education compared to those who were well educated.

### Authors Contribution:

**KH:** Designed the study, Data collection and compilation, Principal investigator, interpretation of data, Manuscript writing, Final approval**.**

**SG:** Data collection, Literature search, Data interpretation, Manuscript writing, Critical revision of article.

**AHA:** Proposed the idea, designed the study, statistical analysis, interpretation of data literature search, supervision of study, reviewed the manuscript and final approval, Accuracy and integrity of the work.
